# Biochemical analysis of some serum trace elements in donkeys and horses in Eastern region of Kingdom of Saudi Arabia

**DOI:** 10.14202/vetworld.2017.1269-1274

**Published:** 2017-10-25

**Authors:** Turke Shawaf, Faisal Almathen, Ahmad Meligy, Wael El-Deeb, Shahab Al-Bulushi

**Affiliations:** 1 Department of Clinical Studies, College of Veterinary Medicine, King Faisal University, 400 Al-Hasa, 31982, Saudi Arabia; 2 Department of Veterinary Public Health and Animal Husbandry, College of Veterinary Medicine, King Faisal University, 400 Al-Hasa, 31982, Saudi Arabia; 3 Department of Physiology, Agricultural Research Center, Egypt; 4 Department of Veterinary Medicine, Infectious Diseases and Fish Diseases, Faculty of Veterinary Medicine, Mansoura University, Mansoura, Egypt

**Keywords:** donkey, horse, serum, trace elements

## Abstract

**Aim:**

Little is known about the serum levels of trace elements in donkeys and horses in Saudi Arabia. This study aimed to investigate the levels of some trace elements in these two species in the eastern region of Saudi Arabia and to compare the obtained results with the reference values.

**Materials and Methods:**

Seventeen Arabian horses and twenty eight Hassawi donkeys were randomly selected for this study. All of the studied healthy animals were kept under a uniform feeding protocol without any minerals supplementations. Atomic absorption spectrometer is used to estimate the serum concentrations of selenium (Se), manganese (Mn), chromium (Cr), copper (Cu), iron (Fe), and zinc (Zn) in the analyzed samples.

**Results:**

Significant differences between horses and donkeys were observed in three of the studied elements (Se, Mn, and Cr). Statistically significant differences were found in serum Se and Cr between male and female horses. The male donkey showed higher Mn, Cu, and Zn levels than female animals.

**Conclusion:**

The obtained results of trace element levels in serum of Hassawi donkeys and Arabian horses in Saudi Arabia are considered as the first values to be published for these breeds. When compared to other animals, the measured amounts of Se, Mn, Cr, Cu, Fe, and Zn in the serum of horses and donkey are often differed, possibly because of the varying living or feeding conditions. Moreover, there were some differences in some of the trace elements concentrations related to animal’s gender and species (horses and donkeys), which will be considered in the interpretation of the laboratory data.

## Introduction

Minerals play an essential role as structural components of the body and in the activities of enzymes and hormones. They are also considered as important constituents of body fluids and tissues and as regulators of cell replication and its differentiation [1]. Minerals are important for all of the physiological processes in animals body [2]. The micro or trace minerals are required by the body in <100 ppm in the diet, and these elements include copper (Cu), chromium (Cr), iron (Fe), manganese (Mn), selenium (Se), and zinc (Zn) [1]. As trace elements are found in the body in low concentrations, any increase or the decrease in their concentrations will be harmful [3]. Trace elements disorders considered as one of the commonly diagnosed problems [4]. However, sometimes trace elements deficiencies can occur without any clinical signs [3]. The clinical interest in trace elements determination for the diagnosis of different diseases has increased in the recent years. Trace elements metabolism may be concerned with the intake, dietary availability, distribution, absorption, excretion, mobilization, storage, and biochemical activity [5]. Results of most trace elements levels for plasma and serum have been assumed to be the similar [6]. Blood is the biological material that is mostly used for the analyses of trace elements due to the level of macro- and micro-elements in the serum which reflects their content in the body [7,8]. Se, has an important role as an antioxidant which is traditionally considered and supplemental Se may have a protective role in animals [9]. It is also considered as a cofactor in metalloenzymes such as glutathione peroxidase. Furthermore, there is some evidence that Se has a role in the immune responses [10]. Mn has an important role in fats and carbohydrates metabolization and also it is important for cartilage formation by playing a role in the synthesis of chondroitin sulfate. Cr is considered as a biosynthesis of glucose tolerance factor. Its deficiency causes impairment of glucose tolerance while the toxicity results in renal failure and dermatitis [11]. Cu is considered as a cofactor for many different enzymes and as a structural component of other proteins which are important for the immune reactions [12]. Fe is considered as the highest numerous trace elements. It is necessary for electron transfer reactions, cell growth and oxygen binding and transportation [13]. Any decrease or increase in Fe concentrations can cause disturbances in the immune system that is why it is important to maintain its amount in the body [14]. Zn is essential for the regulation of gene expression because it is found in the nucleus of the cells and in some of cell proteins [15]. Zn is a trace element that must be supplied through diet [9]. Severe Zn deficiency can lead to physical growth impairments because of the different organ systems that are affected by its deficiency [16,17].

In Saudi Arabia, there are many breeds of donkeys in which the Hassawi breed is considered as one of the most common breeds that are used by the native people [18]. Recently, the horses and donkeys race events and the medical care for these animals increased dramatically [18]. Due to the limitation in the data that are available regarding Se, Mn, Cr, Cu, Fe, and Zn concentrations in horses and donkeys, further studies are needed to determine the reference values for these species [19].

The determination of trace elements profile is also important to obtain the necessary information regarding animal’s health and also the determination of the increase or decrease in these parameters will be useful in the diagnosis and for the appropriate treatment selection [20,21]. As little is known about the concentration of trace elements in donkeys and horses in Saudi Arabia, this study aimed to investigate the levels of some trace elements in these two species in the eastern region of Saudi Arabia and to compare the obtained results with the reference values.

## Materials and Methods

### Ethical approval

The study was approved for research purpose by the Ethics Committee at King Faisal University in Saudi Arabia.

### Animals

A total of 45 randomly selected physically fit and clinically healthy animals including 17 pure Arabian horses (10 females and 7 males) ranged from 4 to 7 years of age, and their body weight ranged from 280 to 435 kg and 28 Hassawi donkeys (16 of them were females and 12 males) their age ranged from 5 to 14 years and their weight ranged from 145 to 270 kg were studied. Before blood collection, the animals were examined physically and clinically to ensure that they are free from any apparent disorders. All of the studied animals were kept under a uniform feeding protocol in which they were fed on pastures, grains such as barley, oat, and corn and concentrate cubes without any minerals supplementations.

### Sample collection

The blood samples were drawn from the jugular vein of all of the 45 studied animals between April and May 2016 at the early morning into sterilized vacutainer plastic blood collection tubes (Guangzhou Improve Medical, China). Centrifugation at 1500 ×*g* for 20 min is used to separate the plasma, and it was collected in a sterile vial to be preserved at −20°C until the trace element analysis were carried out.

### Estimation of serum trace elements

Concentrations of Se, Mn, Cr, Cu, Fe, and Zn were estimated in all of the serum samples using a Shimadzu AAS 6800 atomic absorption spectrometer (Japan). Flame atomic absorption spectrometry (FAAS) supplied by heated graphite atomizer system as well as deuterium set corrected used in the research. Furthermore, the FAAS, an air/acetylene gas (10:1.5) was used. Flame atomic absorption was used for the analysis of Cu, Fe, Mn, and Cr. However, Se analysis was performed using Graphite furnace atomic absorption spectrometry (GFAAS) through argon being as an inert gas. Samples are injected to the GFAAS and FAAS through Shimadzu ASO-6100 automatic sampler. Mars_Xpress (CEM-USA) Microwave Digestion System was used. All of the digestion procedures were done using Teflon reaction vessels. 5 mL of concentrated nitric acid was used for washing the vessels before each digestion process. Samples quantitative analysis was performed by external calibration. Standard solutions were prepared from 19.6% (w/w) HNO_3_ (like the acidic concentrations in the prepared sample). Eight concentrations in the linear dynamic variety were measured. Calibration curve to each analyst was plotted from the limits of detection. To avoid any errors, a slight instrumental drift is monitored by analyzing calibration standards at regular intervals during analysis alongside samples were taken into account. Full quantitative analysis mode was applied for all of the measurements. For digestion, 0.5 g of each serum sample in triplicate manner was weighted directly into separate digestion vessels, followed by the addition of about 7.0 mL of concentrated nitric acid (HNO_3_) (65%) as well as 2.0 ml of hydrogen peroxide (H_2_O_2_) (30%). The combustion procedure was well optimized. The contents of the tubes were cooled then diluted to 50 mL with Deionized doubly distilled water, and also to be included with the samples during digestion and passed through the same dissolution procedures. Condition of digestion in microwave system was applied according to the study by Soylak *et al*.[22] and Waegeneers *et al*. [23].

### Statistical analysis

For all of the analyzed elements, mean, median, and range were analyzed using the descriptive statistical analysis of Graph Pad Prism 7. Comparison between the two species and the impact of sex elements was analyzed using Student’s *t*-test. Differences between groups were considered significant when p<0.05. In addition, the values normal distribution was evaluated by D’Agostino and Pearson omnibus normality test.

## Results

**Figure-1:**
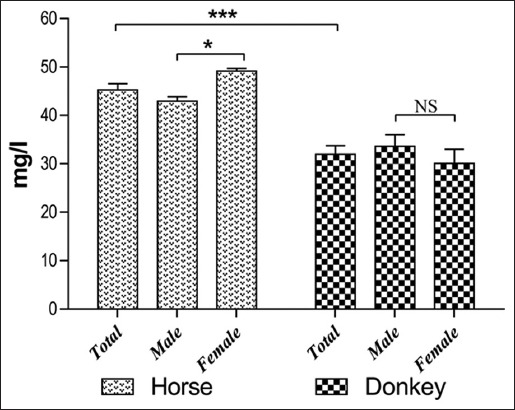
Concentrations (mg/l) selenium in serum of horses and donkeys. NS>0.05, *<0.05, and ***<0.001.

**Figure-2:**
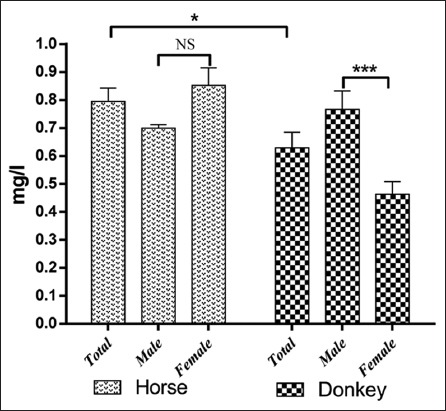
Concentrations (mg/l) manganese in serum of horses and donkeys. NS>0.05, *<0.05, and ***<0.001.

**Figure-3:**
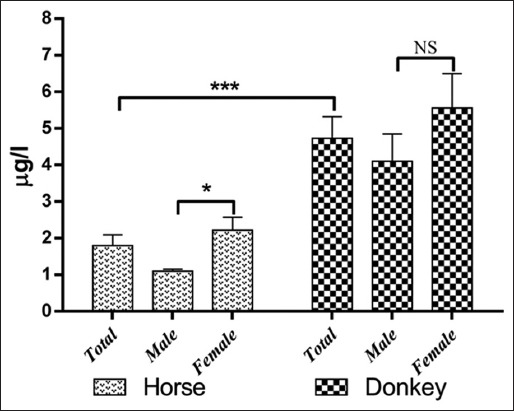
Concentrations (mg/l) chromium in serum of horses and donkeys. NS>0.05 and ***<0.001.

**Figure-4:**
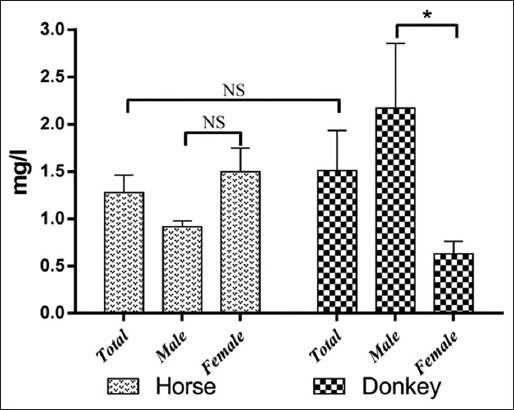
Concentrations (mg/l) copper in serum of horses and donkeys. NS>0.05 and *<0.05.

**Figure-5:**
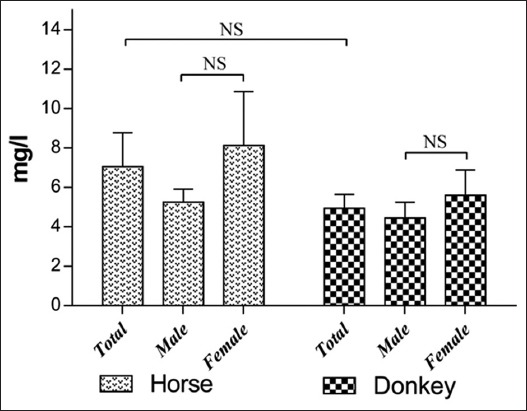
Concentrations (mg/l) iron in serum of horses and donkeys. NS>0.05.

**Figure-6:**
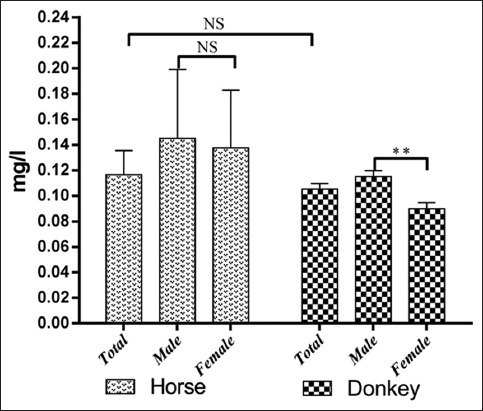
Concentrations (mg/l) zinc in serum of horses and donkeys. NS>0.05 and **<0.01.

The results of serum trace element levels in horses and donkeys and their relation to gender are summarized in Tables-1 and 2 and Figures-1-6. Significant differences between horses and donkeys were observed for three of the studied elements (Se, Mn, and Cr) (Figures-1-3), as serum Se and Mn levels were found to be higher in horses mean±standard error of mean (45.27±1.29) and (0.79±0.05), respectively, compared to that in donkeys which is (33.83±1.71) and (0.636±0.05), respectively. In contrast, the Cr level in horses (1.80±0.3) was significantly lower than that in donkeys (4.73±0.594). However, no statistically significant differences were observed in serum concentrations of Cu, Fe, and Zn between horses and donkeys (Figures-4-6). Interestingly, the range of Cu level was wider in donkeys (1.06-3.12) than that in horses (0.78-1.82). As shown in Table-1, the median value of Fe and Zn levels was similar in horses (5.36 and 0.11, respectively) and donkeys (5.07 and 0.103, respectively). Statistically significant differences were found in serum Se and Cr between male and female horses, in which the concentrations were higher in female than that of male animals as shown in Table-2. When the serum trace elements concentration is compared between the male and female donkeys, male donkeys showed much higher Mn, Cu, and Zn levels than female animals as mentioned in Table-2 and Figures-2,4, and 6. Moreover, female donkeys shown higher levels of Cr and Fe compared to males, but this did not reach statistical significance (Table-2, Figures-3 and 5).

**Table-1:** Descriptive statistics of trace elements concentration in Arabian horses and Hassawi Donkey.

Parameter	Horse (n=17)			Donkey (n=28)		
	
	Mean±SEM	Median	Range	Mean±SEM	Median	Range
Se (mg/l)	45.27±1.29	44.7	41.31-49.12	33.83±1.71	33.67	30.42-35.12
Mn (mg/l)	0.79±0.05	0.74	0.66-0.98	0.636±0.05	0.57	0.36-0.95
Cr (mg/dl)	1.80±0.3	1.53	1.21-2.14	4.73±0.594	4.28	3.98-5.48
Cu (mg/l)	1.28±0.185	1.09	0.78-1.82	1.61±0.426	1.64	1.06-3.12
Fe (mg/l)	7.1±1.73	5.36	4.1-11.88	4.95±0.711	5.07	2.2-8.34
Zn (mg/l)	0.11±0.006	0.11	0.10-0.178	0.104±0.05	0.103	0.064-0.126

Se=Selenium, Mn=Manganese, Cr=Chromium, Cu=Copper, Fe=Iron, Zn=Zinc, SEM=Standard error of mean

**Table-2:** Mean and SEM of trace elements concentration in males and females in both of the Arabian horses and Hassawi Donkeys.

Parameter	Horse (Mean±SEM)		Donkey (Mean±SEM)	
	
	Male (n=7)	Female (n=10)	Male (n=12)	Female (n=16)
Se (mg/l)	42.93±0.97	49.19±0.55*	34.57±1.71	30.09±2.96
Mn (mg/l)	0.71±0.011	0.85±0.06	0.77±0.066	0.463±0.04**
Cr (mg/l)	1.1±0.057	2.22±0.36*	4.10±0.75	5.56±0.94
Cu (mg/l)	0.91±0.067	1.49±0.25	2.17±0.69	0.629±0.13*
Fe (mg/l)	5.25±0.66	8.14±2.74	4.46±0.80	5.60±1.30
Zn (mg/l)	0.147±0.054	0.136±0.036	0.115±0.004	0.090±0.005**

* <0.05,

** <0.01.

Se=Selenium, Mn=Manganese, Cr=Chromium, Cu=Copper, Fe=Iron, Zn=Zinc, SEM=Standard error of mean

## Discussion

Minerals play an essential role as structural components of the body and in the activities of the enzymes and hormones. They are also considered as important constituents of body fluids and tissues and as regulators of cell replication and its differentiation [1]. As only few literatures are available about the trace elements profile in donkeys and horses that are present in the eastern region of Saudi Arabia, this data can be considered as an important reference data, it also indicates the exposure factors of horses and donkeys to some specified elements that are present in the feed or the environment. In this study, the concentrations of trace elements were evaluated in Arabian horses and Hassawi donkey breed. However, the influence of both genders on the levels of trace elements in both species was also analyzed. The results were listed in Figures-1-6 and they were summarized in Tables-1 and 2. Serum is often used to detect the micro- and macro-elements because it reflects their content in the body [7,8].

According to the obtained results that are listed in Table-1, the serum Se concentration was 45.27±1.29 and 33.83±1.71 in Arabian horses and Hassawi donkeys, respectively, which were much higher than the results that are reported by Muirhead *et al*. [24]. In this study, higher Se level was observed in horses when compared to that in donkeys which are agreed with the results reported by Richardson *et al.*, who reported that Se levels in horses are 2.3 times more than that in donkeys serum [25]. The contrary of serum Se to other studies could be attributed to the level of dietary Se intake.

The measured Mn concentrations in donkeys and horses in the current study were more than the data reported for donkeys by Fantuz *et al*. [26] and that for horses by Okumura *et al*. [27]. Moreover, the obtained results of Mn for horses (0.79±0.05) and donkeys (0.636±0.05) were lower than the levels of Mn that are reported in camels by Sharkawy *et al*. [28] which is expected to be due to some factors such as species variations, environmental factors, and dietary factors.

The results of Cr levels in serum for donkeys and horses in the present study were slightly lower than that reported for human by Hasegawa *et al*. [29].

The results of Cu levels in serum for donkeys (1.61±0.426) and horses (1.28±0.185) in the present study were similar to the results reported for donkeys by Fantuz *et al*. [26] and the results for horses reported by Okumura *et al*. [27] and Maia *et al*. [30]. Interestingly, a significant higher level of Cu was found in stallion horses when compared to that of mares (Table-2). According to De Souza *et al*. [31], serum Cu concentrations can be affected by different areas and by the seasonal changes.

Our results did confirm the importance of high Fe levels in the Arabian horses (7.1±1.73 mg/l) and Hassawi donkeys (4.95±0.71 mg/l) than that in Turkman horses (1.56 mg/l) and Turkman donkeys (1.4 mg/l) reported by Zaeemi *et al*. [32]. No significant difference was observed in Fe level between the different genders in both of horses and donkeys (Table-2). Furthermore, similar results were previously reported by Dierenfeld *et al*. [33]. The different results of Fe levels in our study compared to other studies could be attributed to some factors such as breed variations, region, and dietary factors [33].

In our samples, serum Zn levels for donkeys were lower when compared to that reported by Fantuz *et al*. [26] and Zaeemi *et al*. [32]. De Souza *et al*. [31] reported a higher level of Zn in horses than the obtained results. The low concentrations of Zn in both Arabian horses and Hassawi donkeys can be attributed to insufficient mineral supplementation. Furthermore, some other factors can cause variations in Zn concentrations in horses which include environmental factors, dietary changes, and animal’s physiology [34,35].

## Conclusion

The obtained results of trace element levels in serum of Hassawi donkeys and Arabian horses in Saudi Arabia are considered as the first values to be published for these breeds. When compared to other animals, the measured amounts of Se, Mn, Cr, Cu, Fe, and Zn in the serum of horses and donkey are often differed, possibly because of the varying living or feeding conditions. Moreover, there were some differences in some of the trace elements concentrations related to animal’s gender and species (horses and donkeys), which will be considered in the interpretation of the laboratory data.

## Authors’ Contributions

TS participated in the fieldwork, the analysis of data and manuscript drafting, AM carried out the laboratory work and participated in manuscript drafting, WE and FA participated in the fieldwork and drafting of the manuscript, SA participated in the analysis of data, manuscript drafting and revision. All authors read and approved the final manuscript.

## References

[1] Balamurugan B, Ramamoorthy M, Ravi J, Keerthana G, Gopalakrishnan K.M, Kharayat S, Chaudhary G.R, Rahul K (2017). Mineral an important nutrient for efficient reproductive health in dairy cattle. Int. J. Environ. Sci. Technol.

[2] Elrod C.C, Van Amburgh M, Butler W.R (1993). Alterations of pH in response to increased dietary protein in cattle are unique to the uterus. J. Anim. Sci.

[3] Topczewska J (2012). Effects of seasons on the concentration of selected trace elements in horse hair. J. Cent. Eur. Agric.

[4] Humann-Zehank E, Genter M, Hennig-Pauka I, Binder A (2008). Trace mineral status and liver and blood parameters in sheep without mineral supply compared to local roe deer (*Capreolus capreolus*) populations. Small Rumin. Res.

[5] Prashanth L, Kattapagari K.K, Chitturi R, Baddam V, Prasad L (2015). A review on role of essential trace elements in health and disease. J. NTR Univ. Health Sci.

[6] Laven R.A, Livesey C.T, Harmon R.J, Scaletti R (2006). Factors affecting the relationship between caeruloplasmin activity and plasma copper concentration in cattle. Vet. Rec.

[7] Forrer R, Wenker C.H, Gautschi K, Lutz H (2001). Concentration of 17 trace elements in serum and whole blood of plains viscachas (*Lagostomus maximus*) by ICP-MS, their reference ranges, and their relation to cataract. Biol. Trace Elem. Res.

[8] Stanek M, Jaworski Z, Sobotka W, Lipiński K, Olenkowicz R (2016). Influence of an organic supplement of copper, zinc and manganese in feed rations on concentrationsof these elements in the coat of Polish Konik horses. J. Elem.

[9] Ciftci T.U, Ciftci B, Yis O, Guney Y, Bilgihan A, Ogretensoy M (2003). Changes in serum selenium, copper, zinc levels and cu/zn ratio in patients with pulmonary tuberculosis during therapy. Biol. Trace. Elem. Res.

[10] Nève J, Palmieri P (2000). First symposium on human health related aspects of selenium research in Europe. J. Trace Elem. Med. Biol.

[11] Cefalu W.T, Hu F.B (2004). Role of chromium in human health and in diabetes. Diabetes Care.

[12] Schuschke D.A, Nutr J (1997). Dietary copper in the physiology of the microcirculation.

[13] Maggini S, Wintergerst E.S, Beveridge S, Hornig D.H (2007). Selected vitamins and trace elements support immune function by strengthening epithelial barriers and cellular and humoral immune responses. Br. J. Nutr.

[14] Oppenheimer S.J (2001). Iron and its relation to immunity and infectious disease. J. Nutr.

[15] Hambidge M (2000). Human zinc deficiency. J. Nutr.

[16] Caulfield L.E, Zavaleta N, Figueroa A (1999). Adding zinc to prenatal iron and folate supplements improves maternal and neonatal zinc status in a Peruvian population. Am. J. Clin. Nutr.

[17] Fraker P.J, King L.E, Laakko T, Vollmer T.L (2000). The dynamic link between the integrity of the immune system and zinc status. J. Nutr.

[18] Shawaf T, Almathen F, Al-Ahmad J, Elmoslemany A (2016). Morphological characteristics of hassawi donkey, eastern province, Saudi Arabia. Alex J Vet. Sci.

[19] Passlack N, Mainzer B, Lahrssen-Wiederholt M, Schafft H, Palavinskas R, Breithaupt A, Zentek J (2015). Concentrations of strontium, barium, cadmium, copper, zinc, manganese, chromium, antimony, selenium, and lead in the liver and kidneys of dogs according to age, gender, and the occurrence of chronic kidney disease. J. Vet. Sci.

[20] Al-Bulushi S, Shawaf T, Al-Hasani A (2017). Some hematological and biochemical parameters of different goat breeds in Sultanate of Oman “a preliminary study”. Vet. World.

[21] Roubies N, Panousis N, Fytianou A, Katsoulos P.D, Giadinis N, Karatzias H (2006). Effects of age and reproductive stage on certain serum biochemical parameters of chios sheep under greek rearing conditions. J. Vet. Med. A Physiol. Pathol. Clin. Med.

[22] Soylak M, Saracoglu S, Tuzen M, Mendil D (2005). Determination of trace metals in mushroom samples from Kayseri Turkey. Food Chem.

[23] Waegeneers N, Pizzolon J.C, Hoenig M, De Temmerman L (2009). Accumulation of trace elements in cattle from rural and industrial areas in Belgium. Food Addit. Contam.

[24] Muirhead T, Wichtel J, Stryhn H, McClure J (2010). The selenium and Vitamin E status of horses in Prince Edward Island. Can. Vet. J.

[25] Richardson S.M, Siciliano P.D, Engle T.E, Larson C.K, Ward T.L (2006). Effect of selenium supplementation and source on the selenium status of horses. J. Anim. Sci.

[26] Fantuz F, Ferraro S, Todini L, Mariani P, Piloni R, Salimei E (2013). Essential trace elements in milk and blood serum of lactating donkeys as affected by lactation stage and dietary supplementation with trace elements. Animal.

[27] Okumura M, Asano M, Tagami M, Tsukiyama K, Fujinaga T (1998). Serum copper and ceruloplasmin activity at the early growing stage in foals. Can. J. Vet. Res.

[28] Sharkawy A.A, Rateb H.Z, Abdel-Mohsen M (2002). Evaluation of some heavy metals in blood and tissues of male camels as indicator of environmental pollution and its relation to age. Assuit Univ. Bull. Environ. Res.

[29] Hasegawa M, Yoshida K, Wakabayashi H, Sudo A (2012). Cobalt and chromium ion release after large-diameter metal-on-metal total hip arthroplasty. J. Arthroplasty.

[30] Maia L, Souza M.V, Fernandes R.B.A, Fontes M.P.F, Viana M.W.S, Luz W.V (2006). Heavy metals in the horse blood, serum, and feed in Minas Gerais. J Equine Vet. Sci.

[31] De Souza M, Paulo M, Fontes F, Bragança R, Fernandes A (2014b). Heavy metals in equine biological components. R Bras. Zootec.

[32] Zaeemi M, Razmi G.R, Mohammadi G.R, Abedi V, Yaghfoori S (2016). Evaluation of serum biochemical profile in Turkoman horses and donkeys infected with Theileria equi. Rev. Méd. Vét.

[33] Dierenfeld S, Shirley A. N, Craig A, Karen C, Walker W, Jurgen S, Marcus C (2005). Mineral concentrations in serum/plasma and liver tissue of captive and free-ranging Rhinoceros Species. Zoo Biol.

[34] Auer D.E, Ng J.C, Seawright A.A (1988). Assessment of copper and zinc status of farm horses and training Thoroughbreds in South-East Queensland. Aust. Vet. J.

[35] Birick H, Ocal N, Gucus A.I, Ediz B, Uzman M (2005). Seasonal changes of some mineral status in mares. J. Equine Vet. Sci.

